# The Fabrication of Large-Area, Uniform Graphene Nanomeshes for High-Speed, Room-Temperature Direct Terahertz Detection

**DOI:** 10.1186/s11671-018-2602-6

**Published:** 2018-07-03

**Authors:** Weiqing Yuan, Min Li, Zhongquan Wen, Yanling Sun, Desheng Ruan, Zhihai Zhang, Gang Chen, Yang Gao

**Affiliations:** 1 College of Optoelectronic Engineering, Key Laboratory of Optoelectronic Technology and Systems, Ministry of Education, Chongqing University, Chongqing, 400044 People’s Republic of China; 2 School of Electronic Information Engineering, Yangtze Normal University, Fuling, 408100 People’s Republic of China; 3 Institute of Electronic Engineering, China Academy of Engineering Physics, Mianyang, 621900 People’s Republic of China

**Keywords:** Graphene nanomesh, Electron beam lithography, Terahertz detection

## Abstract

In recent years, graphene nanomesh (GNM), a material with high flexibility and tunable electronic properties, has attracted considerable attention from researchers due to its wide applications in the fields of nanoscience and nanotechnology. Herein, we have processed large-area, uniform arrays of rectangular graphene nanomesh (r-GNM) and circular graphene nanomesh (c-GNM) with different neck widths by electron beam lithography (EBL). The electronic properties of those high-quality GNM samples have been characterized systematically. Electrical measurements illustrated that top-gated field effect transistors with different neck widths of the GNM possessed different *I*_on_/*I*_off_ ratios. In particular, the devices based on r-GNM with a neck width of 30 nm were found to possess the largest *I*_on_/*I*_off_ ratio of ~ 100, and the band gap of the r-GNM was estimated to be 0.23 eV, which, to the best of authors’ knowledge, is the highest value for graphene ribbons or a GNM with a neck width under 30 nm. Furthermore, the terahertz response of large-area r-GNM devices based on the photoconductive effect was estimated to be 10 mA/W at room temperature. We also explored the practical application of terahertz imaging, showing that the devices can be used in a feasible setting with a response time < 20 ms; this enables accurate and fast imaging of macroscopic samples.

## Background

Graphene, a single layer of an sp^2^-hybridized carbon film, has drawn great attention in the last few years, as it possesses unique optoelectronic properties, such as high carrier mobility, zero band gap, and frequency-independent absorption. These properties facilitate its potential applications in the field of nanoelectronics, nanocomposites, chemical sensors, biosensors, and photodetectors [1–6]. However, the zero energy gap of graphene limits its applications in electronic and photonic devices. Consequently, it is highly desirable to open the energy gap of graphene and in turn improve the *I*_on_/*I*_off_ ratio [7]. It is universally acknowledged that the band gap of graphene can be tuned by various methods, including application of an electric (or magnetic) field to the bilayered graphene [8, 9], chemical doping [10], application of strain [11], and reshaping of the nanostructure of graphene [12–14]. For example, in 2017, Cheng et al. introduced the chemically regulative graphene with incorporated heteroatoms into the honeycomb lattice and demonstrated microstructure-tailored nanosheets (e.g., 0D quantum dots, 1D nanoribbons, and 2D nanomeshes), which enlarged the band gap and induced special chemical and physical properties of graphene, further presenting promising applications in actuators and power generators [15]. However, among all the methods that tuned the band gap of graphene, reshaping the nanostructure of graphene is currently the most convenient way [16], as it minimizes the inherent electronic properties of graphene [17]. The properties of graphene are reshaped when it is scaled to nanostructures, such as a graphene nanoribbon (GNR) [18–20], graphene nanoring, and graphene nanomesh [21–24]. Sun et al. proposed a simple method to open a comparable band gap in graphene by narrowing it down into a GNR and employed it in FETs, achieving large *I*_on_/*I*_off_ ratios of ~ 47 and ~ 105 at room temperature and 5.4 K, respectively [12]. However, the fabrication of long, narrow GNRs is difficult, which will be an obstacle in the application of nanoelectronic devices. Graphene nanomesh (GNM), a simpler nanostructure to fabricate, can open up a band gap in large graphene sheets, and the FETs based on GNMs can support currents nearly 100 times greater than individual GNR devices [25]. In 2017, Yang et al. utilized a mesoporous silica (meso-SiO_2_) template for the preparation of GNM FETs with improved on/off ratios, constructing highly sensitive biosensors for selective detection of human epidermal growth factor receptor 2. This further proved that it is an effective method to tailor the graphene into the GNM to open the band gap [26]. In general, GNMs can be fabricated by nanoimprint lithography, template-assisted lithography technology, and self-organized growth [27]. Haghiri’s group reported the fabrication of a large surface GNM applied to label-free DNA detection by nanoimprint lithography [22]. Nevertheless, the neck width of the GNM was too large (~ 260 nm) to open the energy gap. Zang et al. demonstrated a novel template-assisted method to prepare GNM using an anodic aluminum oxide membrane as a pattern mask with the help of O_2_ plasma etching [28]. Most of the GNMs are prepared by prefabricating a nanostructured template or nanoparticles as a protective mask for reshaping the graphene layer. However, the synthesis of the nanomask is relatively complex, and the neck width of the GNM is difficult to control to realize the fabrication of large-scale, uniform arrays.

Herein, large-scale, uniform arrays of rectangular graphene nanomeshes (r-GNMs) and circular graphene nanomeshes (c-GNMs) with different neck widths were successfully patterned by electron beam lithography (EBL). In addition, GNM-based terahertz detectors upon the foundation of the photoconductive effect of graphene were fabricated. Electrical measurements were performed at room temperature to gain further insight into the effect of neck width in our GNMs on the performance of the detectors, which illustrated that devices with different neck widths of the GNM possessed different *I*_on_/*I*_off_ ratios and band gaps. It was noted that the current of c-GNM-based devices was larger than that of the r-GNM-based devices while the *I*_on_/*I*_off_ current ratio was smaller; this might be attributed to more edge roughness in r-GNM. Afterwards, the terahertz photocurrents of r-GNM devices with different sizes were also measured, demonstrating the photoconductive effect of this novel structure. Finally, the application of terahertz imaging based on the r-GNM devices using a bifocal imaging system was demonstrated.

### Experimental Section

#### Fabrication of Detectors

**Fig. 1:**
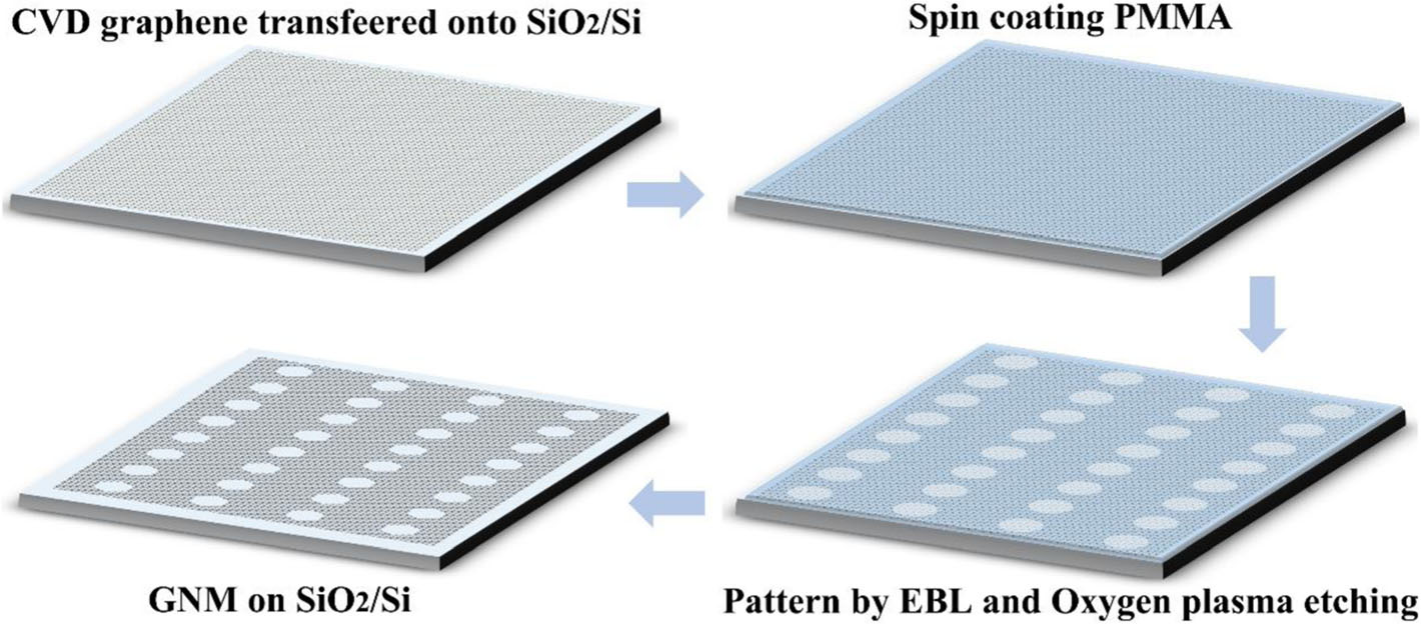
Illustration of the fabrication process of a GNM by EBL

Large-area single-layer graphene was grown by chemical vapor deposition on a copper substrate. It was then transferred onto heavily doped *p*-type Si substrates with a 285-nm SiO_2_ layer using polymethyl methacrylate (PMMA)-assisted wet-transfer techniques [29]. Source and drain electrodes (50-nm-thick Au) were deposited over the graphene through electron beam evaporation followed by a standard metal lift-off technique. The separation distance between the two electrodes was 14 μm. In the second step, we utilized EBL technology to fabricate two kinds of nanomesh graphene: r-GNM and c-GNM. The EBL fabrication route of r-GNM and c-GNM is illustrated in Fig. 1. After the transfer of graphene onto the substrate, the positive e-beam resist, PMMA, was then spun onto the graphene sample and patterned to form an etching mask. The desired shape and size can be determined by the mask. After that, the graphene exposed to air was etched away using oxygen plasma at 5 Pa and 100 W for 5 s. Then, a solution of isopropanol to methyl isobutyl ketone (3:1) was utilized to etch the PMMA away, followed by the deposition of silicon nitride (Si_3_N_4_) gate dielectrics by plasma-enhanced chemical vapor deposition (PECVD). Finally, the gate electrode was deposited over the Si_3_N_4_ through an electron beam evaporation method.

#### Sample Analysis

The morphology and structure of the synthesized r-GNM and c-GNM were characterized by scanning electron microscopy (Hitachi, S-4800). The electrical properties of the detectors were characterized by a semiconductor parameter analyzer (Agilent, 4294A) at room temperature, while the optical characteristics of the devices were tested by the homemade optical measurement system.

## Results and Discussion

**Fig. 2:**
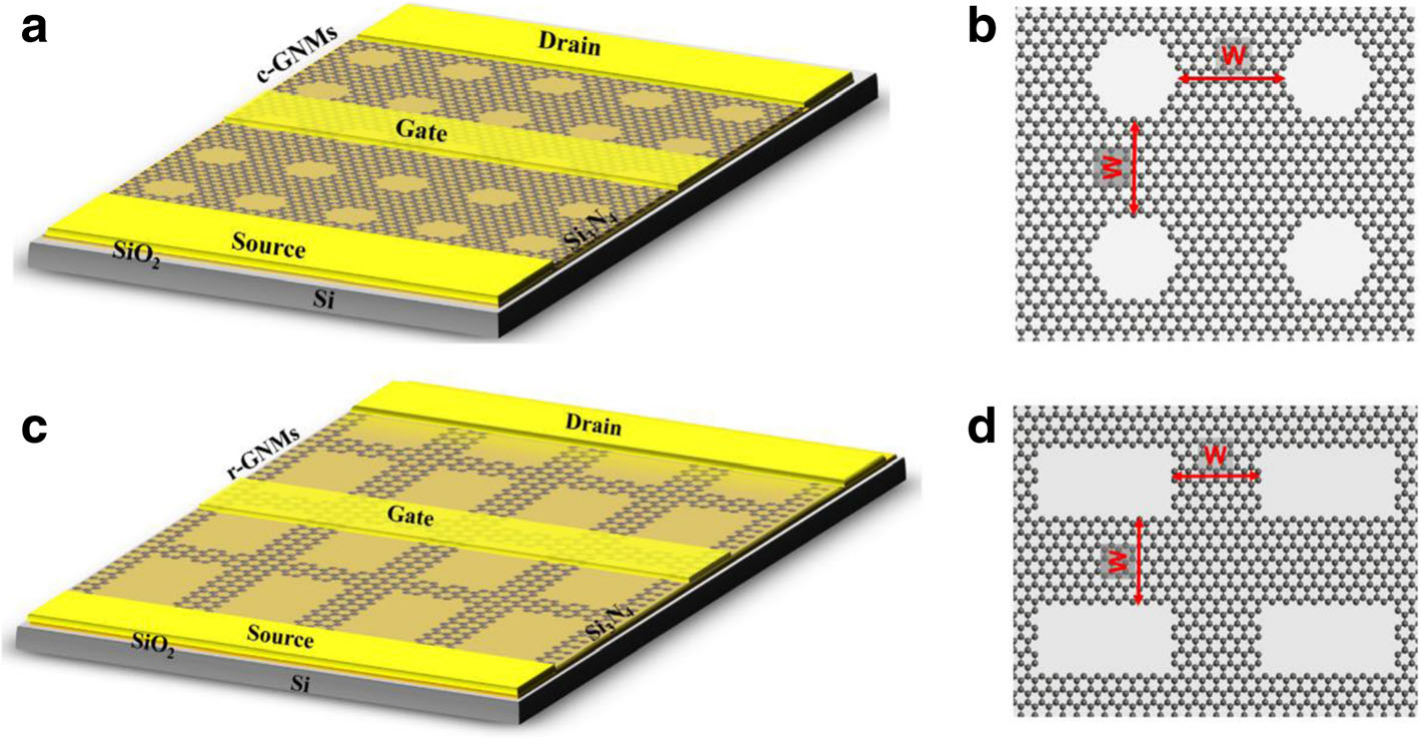
**a** Schematic illustration of the fabricated terahertz detectors based on c-GNM. **b** The structural schematic of c-GNM, where *W* is the neck width. **c** Schematic illustration of the fabricated terahertz detectors based on r-GNM. **d** The structural schematic of r-GNM

A schematic illustration of the fabricated terahertz detectors based on c-GNMs is depicted in Fig. 2a. The source and drain electrodes were deposited on the SiO_2_/Si substrate with the single-layer graphene that was cut out of the c-GNM. The typical geometrical structure of the c-GNM is shown in Fig. 2b. The continuous large-area GNMs with lengths of 20 μm and widths of 60 μm were used as the channel. As graphene is a single layer of atomic structure, in order to reduce the damage in the production of the oxide layer, we choose the silicon nitride (Si_3_N_4_) low-temperature PECVD process to make the dielectric layer. An additional advantage of silicon nitride insulators over silicon oxide for graphene devices is their higher surface polar optical phonon frequency ∼ 110 versus ∼ 56 meV for silicon oxide, which should decrease the importance of remote inelastic phonon scattering in the graphene channel [30]. To further investigate the devices with different nanostructures, the r-GNR-based terahertz detectors were also prepared, and the schematic illustration is given in Fig. 2c. “*W*” in Fig. 2b, d are the neck width values, defined as the minimum distance between the most adjacent nanoholes, which is the most critical parameter in the GNM.

**Fig. 3:**
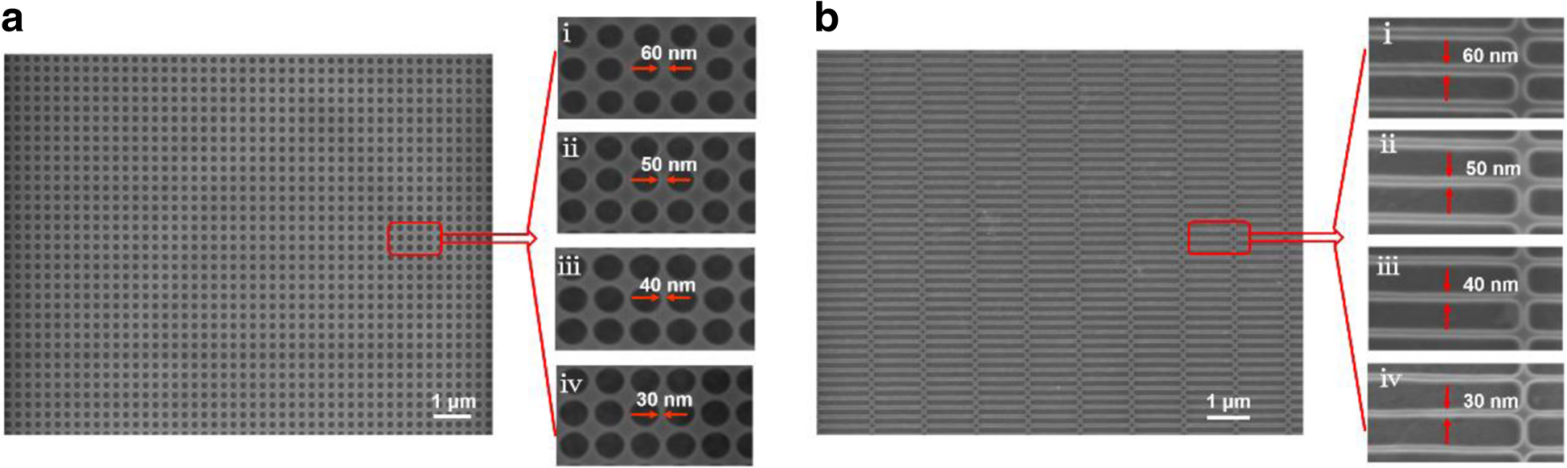
**a** SEM images of c-GNMs with neck widths of (i) 60, (ii) 40, (iii) 50, and (iv) 30 nm. **b** SEM images of r-GNMs with neck widths of (i) 60, (ii) 40, (iii) 50, and (iv) 30 nm

Electrical measurements were performed at room temperature to gain further insight into the effect of neck width in our GNM on the performance of detectors. Herein, four r-GNM and c-GNM arrays with neck widths of 30, 40, 50, and 60 nm, respectively, were patterned by EBL. Figure 3a presents the SEM images of r-GNMs with various neck widths. Figure 3b illustrates the c-GNMs with various neck widths. In this work, the neck width of the GNM is consistent with the layout design by controlling the etching time and the etching power. During the focusing of SEM photos, the scanning electron has a certain effect on the graphene, which leads to the difference in the SEM image color of graphene, but the mesh morphology and size of graphene nanomesh will not be affected. As these images clearly show, both c-GNM arrays and r-GNM arrays could be fabricated uniformly on a large scale using EBL.

To investigate the electronic properties of the GNM, FET devices based on the GNMs with neck widths of 30, 40, 50, and 60 nm were fabricated, respectively. To some extent, the GNM could be regarded as a highly interconnected network of GNRs, and both theoretical and experimental work has shown that the size of the conduction band gap is inversely proportional to the ribbon width. That is, a narrower neck width will gain sufficient band gap energy for sufficient gate response and an on–off ratio, and a denser mesh structure could enable higher current delivery [25].

**Fig. 4:**
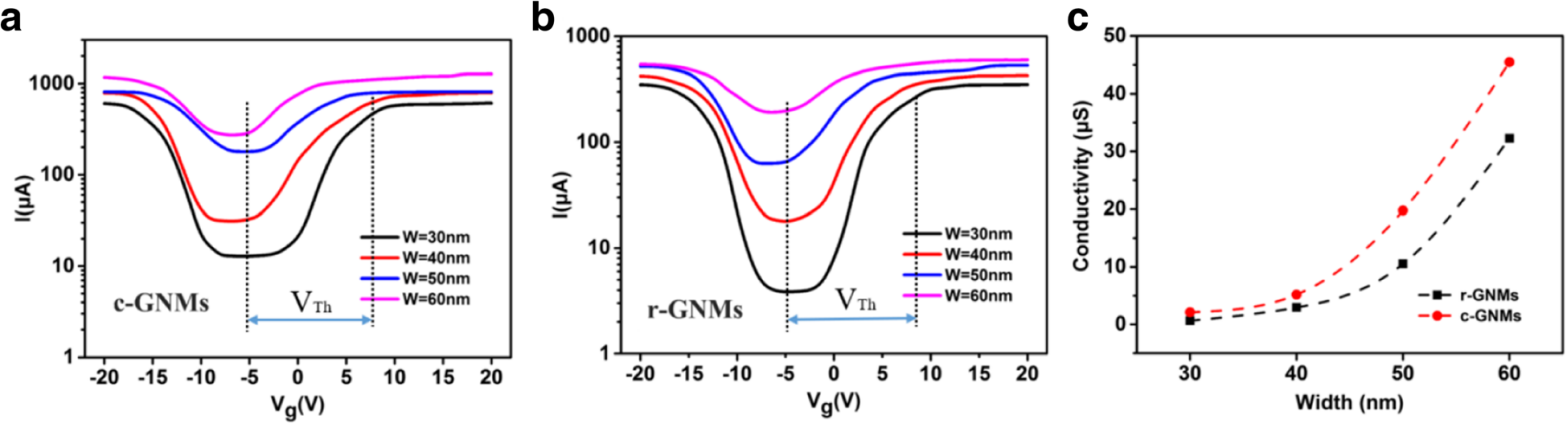
Transfer characteristics (*I*_ds_ − *V*_g_) of the devices based on **a** c-GNM and **b** r-GNM with different widths at *V*_ds_ = 2 V. The *V*_Th_ (the conduction voltage value minus the voltage value of neutral point) of the 30-nm device is about 15 V. **c** Conductivity versus the neck width for r-GNM (black) and c-GNM (red)

Figure 4a shows the transfer characteristics at *V*_ds_ = 2 V for the devices based on c-GNMs with different neck widths of 30, 40, 50, and 60 nm, from which we could determine the corresponding *I*_on_/*I*_off_ ratios of ~ 40, ~ 25, ~ 5, and ~ 4, respectively. The transfer characteristics for the devices based on r-GNMs with different neck widths of 30, 40, 50, and 60 nm are presented in Fig. 4b. Comparing Fig. 4a, b, we can see that the conduction current of c-GNMs is much larger than that of r-GNMs (about two times). As a result of GNM can be viewed as an interconnected network structure of graphene, the actual area of c-GNM delivering current is greater than that of r-GNM, this leads to the current of c-GNM greater than r-GNM under the same conditions. Additionally, the *I*_on_/*I*_off_ ratios of r-GNMs with different neck widths of 30, 40, 50, and 60 nm obtained were ~ 100, ~ 25, ~ 8, and ~ 3, respectively, indicating that the *I*_on_/*I*_off_ ratio of the GNM-based devices can be readily tuned by varying the neck width, which plays an important role in charge transport properties. It was observed that the GNM-based devices in this letter possessed higher *I*_on_/*I*_off_ ratios than many other GNR-based devices with smaller widths [17]. Since the GNM can be considered an interconnected net of GNRs, the generation of the band gap is also due to multiple factors, including lateral quantum confinement [31] in the transmission direction and a Coulomb blockade [32] resulting from the edge defect or roughness [33]. Such a large *I*_on_/*I*_off_ current ratio may result from the long channel effect: the net structures of the GNMs increased the conduction channel of the device, the boundary of the internal nanoholes enhanced quantum-confinement [34], and localization effects were caused by edge defects, such as edge disorder [35] and/or species absorbed on the carbon dangling *π*-bonds in the internal nanoholes [36, 37]. The internal boundary of the r-GNMs is much larger than that of the c-GNMs due to the different geometries. In addition, the circular edge of the c-GNM possesses more defects, making the lateral quantum confinement more remarkable to increase the band gap. These can also explain why the *I*_on_/*I*_off_ current ratio of the r-GNMs is larger than that of the c-GNM. From Fig. 4a, b, it is determined that the devices based on r-GNM and c-GNM exhibited a clear conductance with a minimum value corresponding to the Dirac point at approximately − 5 V. The threshold voltage is obtained by using the voltage at conduction time minus the neutral point voltage. From Fig. 4a, b, we can see that the threshold voltage of the device is about 15 V for 30-nm-size c-GNM and r-GMN. The homologous conductivity obtained is displayed in Fig. 4c. The electrodes of the device are made directly on the original graphene. Only the graphene between the channels is made into nanomesh, and the contact resistance between the metal electrode and the underneath of the semimetal pristine graphene is relatively small. The channel resistance is mainly the resistance of the graphene nanomesh. Owing to a larger area duty ratio at the same area of the conductive channel, the conductivities of the c-GNM-based devices were found to be higher than those of the r-GNM-based devices. Compared to GNRs [38] or other GNMs [39] that were reported before, our c-GNM and r-GNM samples can deliver a higher current owing to their large area and uniform size.

**Fig. 5:**
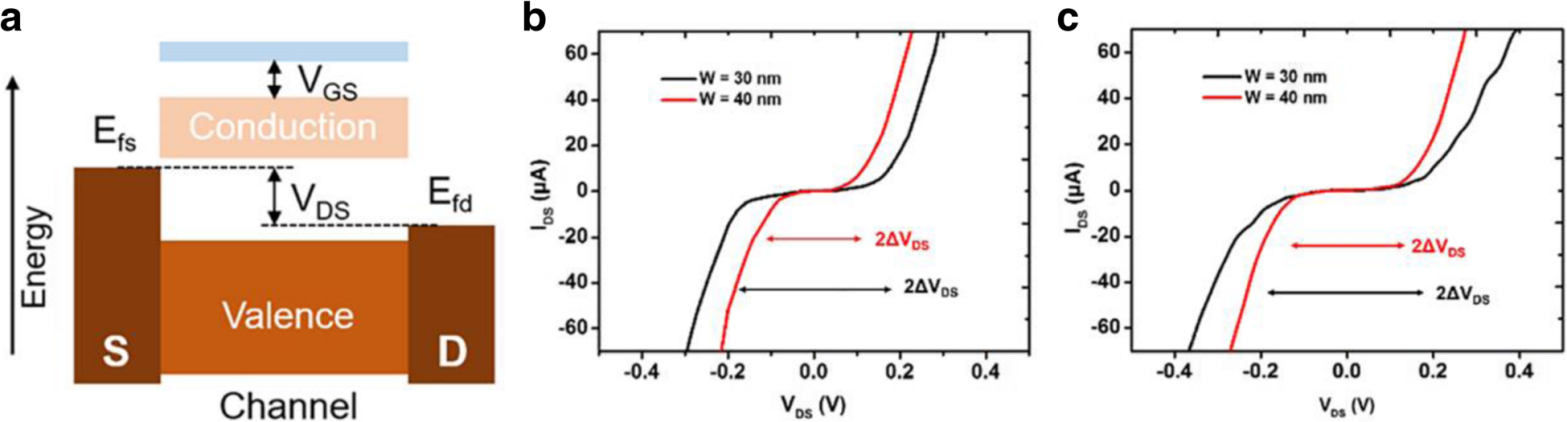
**a** Schematic energy band diagram for a GNR with source and drain electrodes. Curves of *I*_DS_ versus *V*_DS_ at a *V*_GS_ bias near the charge neutral voltage for **b** r-GNM and **c** c-GNM

Figure 5a shows the schematic energy band diagram for GNRs with source and drain electrodes. The source and drain levels approach the conduction and valence band edges, respectively, with an increase in the source–drain voltage (*V*_DS_). When the conduction (valence) band edge falls into the bias window between the source and drain electrodes, electrons (holes) are injected from source (drain), and the current *I* rises sharply. The gate voltage adjusts the position of the gap relative to the source–drain levels. Curves of *I*_DS_ versus *V*_DS_ at a *V*_GS_ bias near the charge neutral voltage for r-GNMs and c-GNMs with neck widths of 30 and 40 nm are illustrated in Fig. 5b, c, which clearly show the “turn-on” and “turn-off” regions, depending on the location of Fermi level. With an increase in the neck width of the GNM, the size of the low-conductance window decreased. For the r-GNMs with widths of 30 and 40 nm, the energy gaps were estimated to be 0.23 and 0.17 eV, respectively (Fig. 5b). Figure 5c illustrates the energy band gap to be 0.19 and 0.16 eV for the c-GNM with widths of 30 and 40 nm, respectively. These values suggest that the band gap was inversely proportional to the neck widths of the GNM channels, and the existence of more edge defects in r-GNM can improve the band gap [23].

**Fig. 6:**
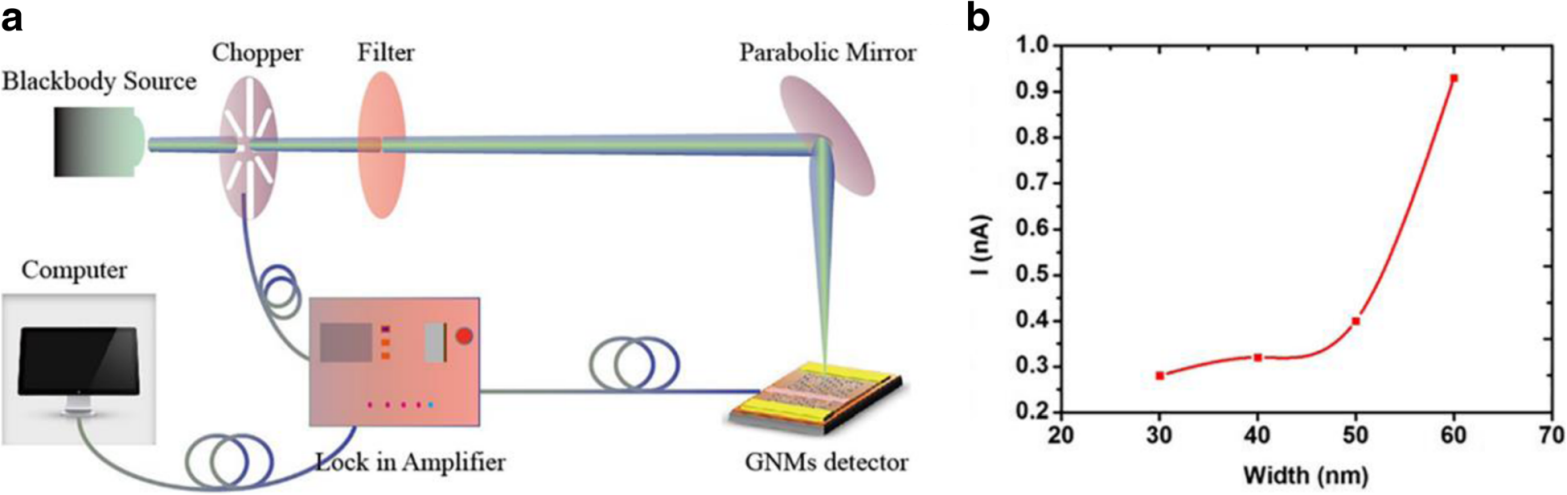
**a** Schematic diagram of the terahertz test experimental setup. **b** Curves of photocurrent *I*_Ph_ versus neck width of r-GNMs

Additionally, the optoelectronic properties of r-GNM devices were investigated by the optical system presented in Fig. 6a to perform photocurrent tests for the r-GNM. In the system, a blackbody source with a 3-THz band-pass filter was used to generate the terahertz radiation, and we measured the *a.c.* photocurrent amplitudes that were obtained using a lock-in amplifier referenced to the chopping frequency. Photocurrent amplitudes were found to be nearly zero without applying a source–drain bias voltage. Owing to the direct contact of the metal electrode and graphene, the photocurrent of photocarriers generated by radiation was relatively weak and counteracted with each other, resulting in an external photocurrent of almost zero.

In addition, the electron–hole pairs generated in the GNM would normally recombine in extremely reduced time, having no contribution to the photocurrent. Therefore, the detection photocurrent existed with an external voltage to separate the photogenerated electron–hole pairs before they recombined. In the investigation reported here, a source–drain voltage of 0.2 V was applied, and photocurrents of 0.28, 0.32, 0.4, and 0.93 nA were obtained under 3 THz radiation, as shown in Fig. 6b, corresponding to different r-GNM devices with neck widths of 30, 40, 50, and 60 nm, respectively. Notably, the photocurrent increased sharply to 0.93 from 0.4 nA. As previously reported, the absorption rate of graphene in visible light is approximately 2.3%, which can be regarded as the thermoelectric effect [40]. While under irradiation of electromagnetic waves with energy below the IR, thermal heating of graphene caused by laser absorption was found to decrease the conductivity of graphene, which contributed to the reason why thermal effects were excluded as the cause of the increased photocurrent of graphene when illuminated. Photoconductive effects mean that when the incident photon energy matches with the energy gap of the GNMs, the energy gap may induce enhanced separation of photon-induced excitons and higher carrier extraction efficiency so that the photocurrent value increases sharply at neck width of 60 nm.

A Golay cell detector (TYDEX GC-1P) was employed to calibrate the light source power to obtain the terahertz responsivity of our GNM-based devices. The responsivity of the r-GNM devices with a neck width 60 nm was found to be 12 mA/W at room temperature.

**Fig. 7:**
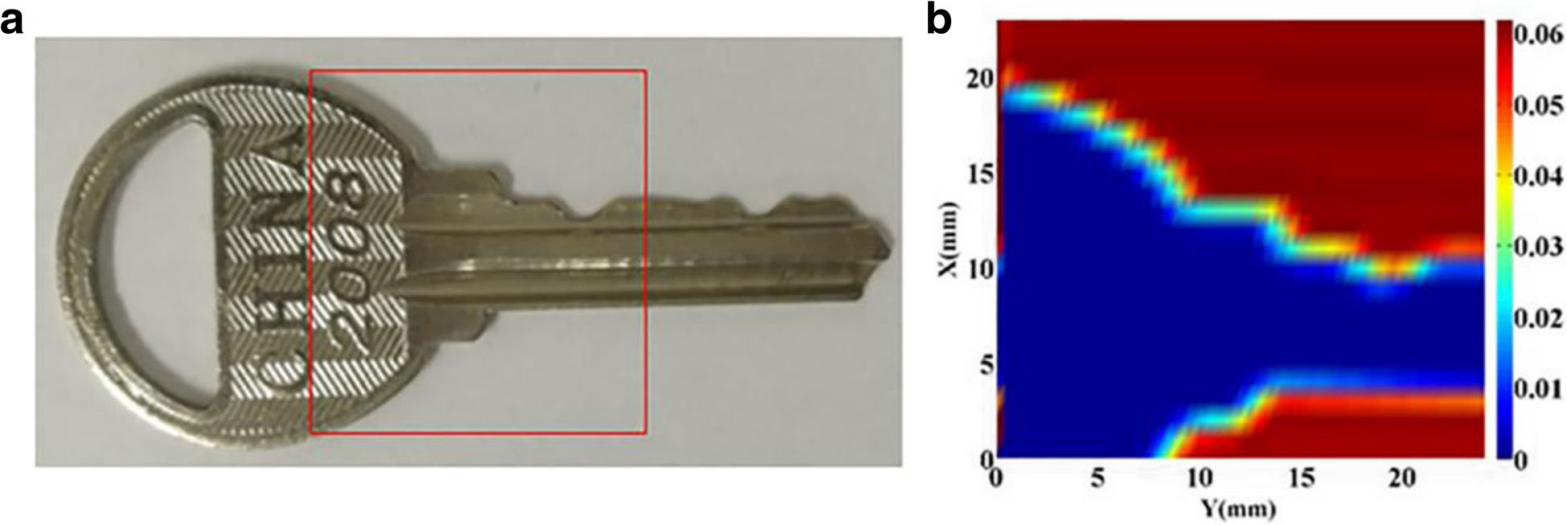
Comparison of a metal key between **a** the optical picture and **b** the terahertz image

Furthermore, the imaging test of the key sample was successfully realized by putting the sample in a simple dual focus imaging system. Due to the maximum movement limit (25 mm × 25 mm) of the nanopositioning system, the terahertz image of one part was obtained, as illustrated in Fig. 7, clearly showing the profile of the key sample. Further, the terahertz image of the key sample was finished by continuously scanning 50 × 50 points with a total time of approximately 75 s, in which the response time for one single detection is less than 20 ms. This work demonstrates that our r-GNM device can be used as a terahertz detector for accurate and fast imaging of macroscopic samples.

## Conclusions

In conclusion, top-gated FETs employing large-area arrays of ordered r-GNM and c-GNM with different neck widths were successfully processed by EBL. The top-gated FETs were fabricated with continuous GNM as the conductive channel. At room temperature, the electrical measurements were performed, which illustrated that the devices with different neck widths of the GNM possessed different *I*_on_/*I*_off_ ratios and energy gaps. Especially, the devices based on r-GNM with a neck width of 30 nm were found to possess the largest *I*_on_/*I*_off_ ratio, ~ 100, and the energy gap was estimated to be 0.23 eV. Though the current of the c-GNM-based devices was larger than that of the r-GNM-based devices, the *I*_on_/*I*_off_ current ratio was smaller, which may be due to the larger edge roughness in the r-GNM. Furthermore, based on the photoconductive effect, the terahertz response of the r-GNM-based device was measured to be 10 mA/W. For practical applications of the devices, a terahertz imaging experiment was performed at room temperature. It was found that such devices can be applied in accurate and fast imaging of macroscopic samples.

## Abbreviations

c-GNM: Circular graphene nanomesh
EBL: Electron beam lithography
FETs: Field-effect transistors
GNM: Graphene nanomesh
GNR: Graphene nanoribbon
MIBK: Methyl isobutyl ketone
PECVD: Plasma-enhanced chemical vapor deposition
PMMA: Polymethyl methacrylate
r-GNM: Rectangular graphene nanomesh

## References

[1] Novoselov KS, Geim AK, Morozov SV, Jiang D, Zhang Y, Dubonos SV, Grigorieva IV, Firsov AA (2004). Electric field effect in atomically thin carbon films. Science.

[2] Geim AK, Novoselov KS (2007). The rise of graphene. Nat Mater.

[3] Heerema SJ, Dekker C (2016). Graphene nanodevices for DNA sequencing. Nat Nanotechnol.

[4] Li J, Niu L, Zheng Z, Yan F (2014). Photosensitive graphene transistors. Adv Mater.

[5] Zhang R, Wei C (2017). Recent advances in graphene-based nanomaterials for fabricating electrochemical hydrogen peroxide sensors. Biosens Bioelectron.

[6] Bhattacharya S, Saha D, Bid A, Mahapatra S (2014). A continuous electrical conductivity model for monolayer graphene from near intrinsic to far extrinsic region. IEEE Trans Elect Dev.

[7] Xia F, Farmer DB, Lin Y, Avouris P (2010). Graphene field-effect transistors with high on/off current ratio and large transport band gap at room temperature. Nano Lett.

[8] Zhang Y (2009). Direct observation of a widely tunable bandgap in bilayer graphene. Nature.

[9] Samarakoon DK, Wang X (2010). Tunable band gap in hydrogenated bilayer graphene. ACS Nano.

[10] Yu WJ, Liao L, Chae SH, Lee YH, Duan X (2011). Toward tunable band gap and tunable Dirac point in bilayer graphene with molecular doping. Nano Lett.

[11] Chen C, Wu JZ, Lam KT, Hong G, Gong M, Zhang B, Lu Y, Antaris AL, Diao S, Guo J (2015). Graphene nanoribbons under mechanical strain. Adv Mater.

[12] Sun J, Iwasaki T, Muruganathan M, Mizuta H (2015) Lateral plasma etching enhanced on/off ratio in graphene nanoribbon field-effect transistor. Appl Phys Lett 106:033509.

[13] Berrada S, Nguyen VH, Querlioz D, Saint-Martin J, Alarcón A, Chassat C, Bneourl A, Dollfus P (2013) Graphene nanomesh transistor with high on/off ratio and good saturation behavior. Appl Phys Lett 103:183509.

[14] Ozyilmaz B, Jarillo-Herrero P, Efetov D, Kim P (2007). Electronic transport in locally gated graphene nanoconstrictions. Appl Phys Lett.

[15] CCheng H, Huang Y, Shi G, Jiang L, Qu L (2017) Graphene-based functional architectures: sheets regulation and macrostructure construction toward actuators and power generators. Acc Chem Res 50:1663–1671. 10.1021/acs.accounts.7b00131 28657710

[16] Wang L, Msh B, Kidambi PR, Jang D, Hadjiconstantinou NG, Karnik R (2017). Fundamental transport mechanisms, fabrication and potential applications of nanoporous atomically thin membranes. Nat Nanotechnol.

[17] Marmolejo-Tejada JM, Velasco-Medina J (2016). Review on graphene nanoribbon devices for logic applications. Microelectron J.

[18] Son YW, Cohen ML, Louie SG (2006). Energy gaps in graphene nanoribbons. Phys Rev Lett.

[19] Chen YC, Cao T, Chen C, Pedramrazi Z, Haberer D, de Oteyza DG, Fischer FR, Louie SG, Crommie MF (2015). Molecular bandgap engineering of bottom-up synthesized graphene nanoribbon heterojunctions. Nat Nanotechnol.

[20] Solís-Fernández P, Bissett MA, Tsuji M, Ago H (2015). Tunable doping of graphene nanoribbon arrays by chemical functionalization. Nanoscale.

[21] Jun Y, Mingze M, Laiquan L, Yufei Z, Wei H, Xiaochen D (2014). Graphene nanomesh: new versatile materials. Nanoscale.

[22] Zribi B, Castro-Arias JM, Decanini D, Gogneau N, Dragoe D, Cattoni A, Ouerghi A, Korri-Youssoufi H, Haghiri-Gosnet AM (2016). Large area graphene nanomesh: an artificial platform for edge-electrochemical biosensing at the sub-attomolar level. Nanoscale.

[23] Tang G, Zhang Z, Deng X, Fan Z, Zeng Y, Zhou J (2014). Improved scaling rules for bandgaps in graphene nanomeshs. Carbon.

[24] Özyilmaz BB et al (2007) Electronic transport in locally gated graphene nanoconstrictions. Appl Phys Lett 91:192107.

[25] Bai J, Zhong X, Jiang S, Huang Y, Duan X (2010). Graphene nanomesh. Nat Nanotechnol.

[26] Yang Y, Yang X, Zou X, Wu S, Wan D, Cao A, Liao L, Yuan Q, Duan X (2017) Ultrafine graphene nanomesh with large on/off ratio for high-performance flexible biosensors. Adv Funct Mater 27:1604096.

[27] König M, Ruhl G, Batke JM, Lemme MC (2016). Self-organized growth of graphene nanomesh with increased gas sensitivity. Nanoscale.

[28] Zeng Z, Huang X, Yin Z, Li H, Chen Y, Li H, Zhang Q, Ma J, Boey F, Zhang H (2012). Fabrication of graphene nanomesh by using an anodic aluminum oxide membrane as a template. Adv Mater.

[29] Li X, Cai W, An J, Kim S, Nah J, Yang D, Piner R, Velamakanni A, Jung I, Tutuc E (2009). Large-area synthesis of high-quality and uniform graphene films on copper foils. Science.

[30] Zhu W, Neumayer D, Perebeinos V, Avouris P (2010). Silicon nitride gate dielectrics and band gap engineering in graphene layers. Nano Lett.

[31] Barone V, Hod O, Scuseria GE (2006). Electronic structure and stability of semiconducting graphene nanoribbons. Nano Lett.

[32] Han MY, Ozyilmaz B, Zhang Y, Kim P (2007). Energy band-gap engineering of graphene nanoribbons. Phys Rev Lett.

[33] Yuan W, Wen Z, Li M, Chen L, Chen G, Ruan D, Gao Y (2016). Energy gap of novel edge-defected graphene nanoribbons. Jpn J Appl Phys.

[34] Tian W, Zeng YC, Zhang ZH (2013). Electronic properties of graphene nanoribbons with periodically hexagonal nanoholes. J Appl Phys.

[35] Zozoulenko MEAI, Heinzel HXAT (2008). Edge-disorder-induced Anderson localization and conduction gap in graphene nanoribbons. Phys Rev.

[36] Xiang H, Kan E, Wei SH, Whangbo MH, Yang J (2009). “Narrow” graphene nanoribbons made easier by partial hydrogenation. Nano Lett.

[37] Zheng XH, Huang LF, Wang XL, Lan J, Zeng Z (2012). Band gap engineering in armchair-edged graphene nanoribbons by edge dihydrogenation. Comput Mater Sci.

[38] Murali R, Yang Y, Brenner K, Beck T, Meindl JD (2009) Breakdown current density of graphene nanoribbons. Appl Phys Lett 94:243114.

[39] Cho SH, Sun SK, Yi J, Park WI (2016). Chemical and biological sensors based on defect-engineered graphene mesh field-effect transistors. Nano Conv.

[40] Sun Z, Chang H (2014). Graphene and graphene-like two-dimensional materials in photodetection: mechanisms and methodology. ACS Nano.

